# The regulatory role of PDE4B in the progression of inflammatory function study

**DOI:** 10.3389/fphar.2022.982130

**Published:** 2022-10-06

**Authors:** Yue Su, Jiaxiang Ding, Fan Yang, Cuixia He, Yuanyuan Xu, Xingyu Zhu, Huan Zhou, Hongtao Li

**Affiliations:** ^1^ First-in-Human Clinical Trial Wards in the National Institute of Clinical Drug Trials, The First Affiliated Hospital of Bengbu Medical University, Bengbu, China; ^2^ School of Public Foundation, Bengbu Medical University, Bengbu, China; ^3^ Department of Ophthalmology, First Affiliated Hospital of Anhui Medical University, Hefei, China; ^4^ School of Pharmacy, Bengbu Medical University, Bengbu, China

**Keywords:** inflammation, cancer, PDE4B, cellular signaling pathways, CRISPR/Cas9

## Abstract

Inflammation is a response of the body to external stimuli (eg. chemical irritants, bacteria, viruses, etc.), and when the stimuli are persistent, they tend to trigger chronic inflammation. The presence of chronic inflammation is an important component of the tumor microenvironment produced by a variety of inflammatory cells (eg. macrophages, neutrophils, leukocytes, etc.). The relationship between chronic inflammation and cancer development has been widely accepted, and chronic inflammation has been associated with the development of many cancers, including chronic bronchitis and lung cancer, cystitis inducing bladder cancer. Moreover, chronic colorectitis is more likely to develop into colorectal cancer. Therefore, the specific relationship and cellular mechanisms between inflammation and cancer are a hot topic of research. Recent studies have identified phosphodiesterase 4B (PDE4B), a member of the phosphodiesterase (PDEs) protein family, as a major cyclic AMP (cAMP) metabolizing enzyme in inflammatory cells, and the therapeutic role of PDE4B as chronic inflammation, cancer. In this review, we will present the tumors associated with chronic inflammation, and PDE4B potential clinical application.

## 1 Introduction

Inflammation is part of the innate immune response to danger signals, tissue destruction, and/or infection. Short-term and properly terminated inflammation is beneficial, but chronic inflammation increases cancer risk (Greten and Grivennikov, 2019). Cancer is a biologically heterogeneous disease with distinct genetic abnormalities (Kumar and Sharawat, 2018), despite progress across the continuum of cancer research and patient care, remains one of the major diseases affecting human longevity and quality of life (Liu and Shi, 2021). In the Lancet, Gilles Dagenais and colleagues found that cancer is the most common cause of death in high-income countries and several middle-income countries (Dagenais et al., 2020; Siegel et al., 2021). The international agency for research on cancer (IACR) predicted that by 2040, approximately thirty million people worldwide will be living with cancer, 60% of whom will die from cancer or its complications. In addition, the incidence of cancer is likely to be higher in relatively underdeveloped countries (Sengupta and Honey, 2019). Recent studies suggest that the loss of polarity and adhesion of cancer cells is a key reason for the ease of metastasis as well as the proliferation of tumor cells compared to normal cells (Yan et al., 2018), in addition to complex biological pathways and mechanisms that target carcinogenesis and maintain cancer phenotypes multiple studies (Cai et al., 2021). Overall, inflammation recruits a variety of inflammatory cells, induces cell proliferation, leads to DNA damage, and increases the risk of cancer (Coussens and Werb, 2002).

PDE4B is a type IV cAMP -specific cyclic nucleotide PDE family member (Ahmad et al., 2015). The encoded protein regulates the cellular concentration of cyclic nucleotides and thus plays a role in signal transduction of inflammatory factors. Recent studies have shown that PDE4B expression is elevated at the transcriptional as well as the translational level in various cancers (Azevedo et al., 2014). PDE4B is located on chromosome 1p31, informative SNPs in the gene cluster encoding PDE4B are located at the 5′ end of the gene (Bender and Beavo, 2006). The PDE4B gene also encodes PDE4B monomers known as PDE4B2 and PDE4B5 (Campbell et al., 2017). Among all subtypes of PDE4, PDE4B is closely associated with cancer and has a major contribution to the role in hematological malignancies (Bolger, 2017). Aberrant expression of PDE4B was found in multiple organs with inflammation, including hematologic (Jiang et al., 1998; Moon et al., 2002; Nam et al., 2019; Rickles et al., 2010), colorectal (Kim et al., 2019), liver and other organs (Hsien Lai et al., 2020).

This review summarizes the relationship between inflammation and cancer. On this basis, the potential clinical value of PDE4B is also discussed.

## 2 Overview of inflammation in human disease processes

Inflammation is a stress response of the organism in the face of multiple stimuli or infection by foreign substances, such as physical injury, infection-induced cellular changes and immune responses present in many disease processes (Coussens and Werb, 2002), and one of the initiation processes of cell trafficking to the tumor microenvironment by specific cytokines called chemokines, which have an important role in many cellular activities, especially in the immune system (Guo et al., 2013). If the stimulus that induces inflammation is persistent, it predisposes to the development of chronic inflammation. Chronic inflammation results in persistent tissue damage and stimulates cell proliferation and tissue repair. Chronic inflammation is manifested by the release of mononuclear cell infiltration, fibroblast proliferation, and other releases that induce the formation of granulation tissue (Gleeson et al., 2011). Inflammation can trigger tumorigenesis through DNA damage in the absence of any exogenous carcinogens (Meira et al., 2008). It has been shown that mouse models of inflammation-associated tumorigenesis have also been shown to be associated with sporadic tumorigenesis. After external stimulation of the body’s own immune system, macrophages and eosinophils increase, intensifying the oxidative stress process. Cellular signaling pathway triggered by inflammatory cytokines promotes tumor development (McGranahan and Swanton, 2017; Li et al., 2022), and it is clear that chronic inflammation increases cancer risk.

The development of cancer and its prognosis are regulated by inflammation, which can promote or inhibit tumor progression and interfere with tumor treatment (Zhao et al., 2021). Tumor refers to the proliferation of local tissue cells under the action of many carcinogenic factors. In recent decades, finding new therapeutic targets or developing more effective treatment options has been our focus (Vineis and Wild, 2014). In recent years, the causal relationship between inflammation and cancer has gradually been recognized as cancer research has intensified. The hypothesis that chronic inflammation might be the origin of cancer was proposed in the 19th century, the site that triggers chronic inflammation induces excessive cell proliferation (Korniluk et al., 2017). Although only cellular over proliferation does not cause cancer, during the recovery of damaged tissues, various cellular molecules such as, growth factors, activation mechanisms, and DNA are highly activated, while inflammation triggers reactive oxygen/nitrogen species against pathogens that damage DNA and other biomolecules, and intracellular responses to DNA damage promote inflammation, creating positive feedback and interfering with repair mechanisms *in vivo* (Suarez-Carmona et al., 2017). In conclusion persistent infection *in vivo* induces chronic inflammation and increases tumor risk (Singh et al., 2019). Cancer, which refers to a group of the world’s most severe and deadly diagnosed pathophysiological conditions (Diori Karidio and Sanlier, 2021), is a huge public health challenge, which has been further exacerbated by the 2019 novel coronavirus pneumonia (COVID-19) pandemic since March 2020 (Sengupta and Zaidi, 2021; Smith et al., 2021).

Many malignant tumors such as lung cancer (Chen et al., 2021), colorectal cancer (Yashiro, 2014), and prostate cancer (Sfanos et al., 2018) are mostly found in chronic inflammation or infection sites, further demonstrating that persistent inflammation may induce cancer development (Kay et al., 2019). Thus it is necessary to elucidate the interaction between inflammation and cancer, and some biomolecules in cells play an important role in inflammation-induced cancer. For example, PDE4 has recently emerged as a key regulator of carcinogenesis. Studies have shown that PDE4 expression is elevated in various cancer species (Jacob et al., 2002; Nagy et al., 2013; Smith et al., 2005). As one of the four isoforms of PDE4, phosphodiesterase 4A (PDE4A) is associated with expression in various cancers and its involvement in VEGF-mediated angiogenesis accelerates epithelial mesenchymal transformation in cancer (Kolosionek et al., 2009). In addition, phosphodiesterase 4D (PDE4D) stimulates the development of lung cancer through TGF-β1 (He et al., 2014). PDE4B, which was brought to our attention, is also a member of the PDE4 family.

## 3 Overview of phosphodiesterase 4B

**FIGURE 1:**
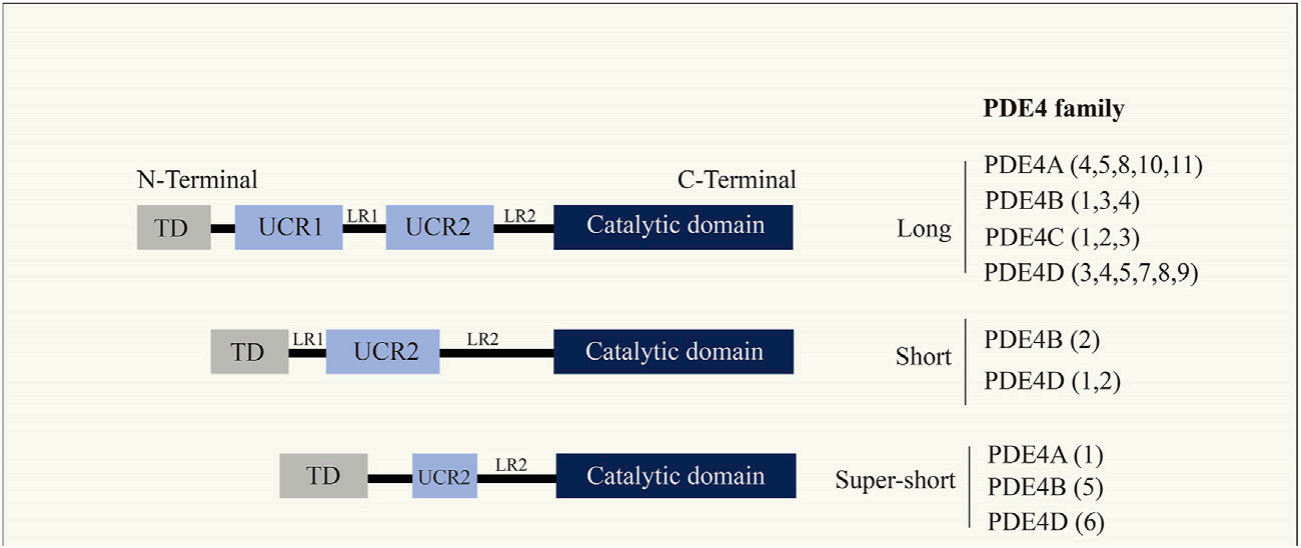
Cyclic nucleotide phosphodiesterase (PDE4) family. PDE4 enzymes are usually divided into four isoforms (PDE4A, PDE4B, PDE4C, PDE4D), where each isoform has multiple transcription products.

PDEs are a diverse family of enzymes that have long been recognized due to their unique tissue distribution, structural and functional properties, and sensitivity to selective inhibitors, considered an attractive and excellent therapeutic target (Azam and Tripuraneni, 2014; Fortin et al., 2009; Komatsu et al., 2013). PDE4B, a member of the PDE family (Figure 1), functions to break down cyclic nucleotides such as cAMP and cyclic guanosine monophosphate (cGMP), thereby reducing the signaling of these important second messengers in cells. cAMP has been considered as an inducer of anti-inflammatory responses, and cAMP-dependent pathways are widely used in pharmacology for the treatment of inflammatory diseases. Recently, cAMP has also been indicated as a coordinator of key steps in the resolution of inflammation (Tavares et al., 2020). Apart from that, cAMP is a secondary messenger responsible for regulating cellular metabolism by activating protein kinase A (PKA) and targeting exchange proteins directly activated by cAMP. cAMP may be involved in controlling a variety of cell functions that are significant in all cell types. It has been shown that PDE4B knockdown effectively inhibit Lipopolysaccharide (LPS)-induced nuclear factor kappa-B (NF-κB) activation and inflammatory responses in multiple cell types, and, PDE4B deletion impairs LPS-induced reactive oxygen species (ROS) generation (Tavares et al., 2020). Analysis of all relevant literature on PDE4B so far revealed that the expression of PDE4B was upregulated in the majority of tumor tissues. Further studies revealed that PDE4B mostly regulates the development of various cancers through the regulation of cAMP. In conclusion, we should pay attention to the regulatory role of PDE4B in tumors.

## 4 Functional regulatory role of phosphodiesterase 4B in human inflammatory diseases

### 4.1 Hematological

The blood system includes several blood-forming organs and various blood cells, and is one of the systems that make up the body. Inflammation, especially chronic inflammation, is an important factor in promoting the development of tumors. At some levels, abnormal peripheral blood counts in the organism due to inflammatory responses are associated with cancer development to some extent (Diakos et al., 2014; Ocana et al., 2017). Some studies have shown that many inflammatory indicators have clinical importance in the prognosis of patients with tumors (Rambaldi et al., 2013; Annibali et al., 2019). Diffuse large B-cell lymphoma (DLBCL) is a common and often fatal malignancy, with an estimated more than 100,000 new cases annually worldwide. Since DLBCL is a genetically heterogeneous disease, the search for new therapeutic targets is important for the treatment of this disease (Schmitz et al., 2018). It is worthy of note that PDE4B is overexpressed in lethal/refractory tumors (Schick and Schlegel, 2022). PDE4B inactivates the second messenger cAMP and abrogates its inhibitory effect in B lymphocytes. cAMP is a ubiquitous second messenger that regulates multiple cellular processes by activating PKA, an exchange protein that is activated directly by cAMP, and other less well-characterized effector proteins following B cell receptor activation, cAMP downregulates signaling pathways responsible for cell proliferation (Kim et al., 2015; Zhang et al., 2020). Since PDE4B terminates cAMP activity, the growth inhibitory effect of cAMP signaling is limited. Thus, DLBCs expressing high PDE4B levels may be resistant to cAMP-induced apoptosis [55, (Kim et al., 2009)]. In summary, PDE4B is an important upstream regulator of cAMP, which provides a new idea for the clinical treatment of DLBCs.

Hany Ariffin et al. (2017) found that the pathogenesis of childhood acute lymphoblastic leukemia (ALL) patients and the prognosis of the organism’s performance profile are biologically similar to the process of accelerated cellular senescence (eg: chronic inflammation as well as telomere depletion). ALL is characterized by an excess of immature lymphocytes, which are more common in children between the ages of 2 and 5 years than in adults (Medinger et al., 2019). Rennan Garcias Moreira et al. (2022) found that PDE4B promotes tumor angiogenesis in ALL. Notably, in Native Americans, the PDE4B allele rs6683977 variant is associated with ALL relapse (Chen et al., 2020). After B cell receptor activation, cAMP downregulated signaling pathways responsible for cell proliferation, and cAMP-mediated life activities were mostly inhibitory, including cell cycle arrest and apoptosis. In conclusion, PDE4B overexpression abrogates cAMP inhibition of cell proliferation (Ahlström et al., 2005; Zhao et al., 2016), providing theoretical support for clinical application in the treatment of ALL.

### 4.2 Colon and rectum

The colon and rectum are important components of the human intestinal tract. Chronic inflammation induced by chronic inflammatory bowel disease or poor dietary habits of organisms are more likely to develop colorectal cancer (CRC) (Schäfer and Werner, 2008)^,^ The main causative factor of colorectal inflammation-induced cancer may be the inflammatory intervention of one or more signaling pathways in regulating tumor progression. CRC is the third most common cancer in both men and women and has one of the lowest survival rates of all cancer types (Biller and Schrag, 2021). Chronic inflammation triggered by external factors may increase the chances of tumorigenesis (Quail and Joyce, 2013). Badar Mahmood et al. (2016) found that lower PDE4 activity detected in functional assays of CRC biopsies contradicted the observations of increased tissue expression and abundance of PDE4B, speculating that CRC disease may produce nonfunctional PDE4B protein with disease-induced frugality. Compensatory mRNA and protein elevations result in higher expression of PDE4B in patients with colorectal tumors. In conclusion, it is necessary to pay attention to the regulatory role played by elevated transcriptional levels of PDE4B in chronic inflammation of the colorectum and colorectal cancer.

### 4.3 Lung

The lung is an important organ of the human body. The unique physiological structure of the lung makes the lung tissue susceptible to bacteriological inflammatory damage when attacked by pathogenic microorganisms, which gradually accumulates and leads to the development of lung cancer (Jacobs and Kligerman, 2019). In response to external stimuli, inflammation in the lung produces excess ROS and chronic inflammation of the lung predisposes to lung cancer (Kachuri et al., 2020). With approximately two million new cases of lung cancer worldwide each year, lung cancer is one of the cancers with a high incidence as well as a high mortality rate worldwide (Thai et al., 2021). With approximately two million new cases of lung cancer worldwide each year, lung cancer is one of the cancers with a high incidence as well as a high mortality rate worldwide (Thai et al., 2021). Miyako Ariga et al. demonstrated that PDE4B regulates neutrophil much more than previously known. Therefore the role of PDE4B in lung cancer cannot be ignored (Jin et al., 2005). Similar to other tumors, PDE4B is also involved in the growth of lung cancer cells through cAMP, and cAMP acts as a second messenger that can regulate cellular responses through activated effectors (Rahamim Ben-Navi et al., 2016; Blommaert et al., 2019). cAMP plays an important regulatory function in almost all cell types involved in the airway pathogenesis of asthma and other chronic inflammatory diseases. Rong-quan He et al. found that the expression of PDE4B is increased in Non-small cell lung cancer (NSCLC), proving that PDE4B has cytotoxicity in lung cancer cells. The best known effector of cAMP is PKA. Shaikh et al. (2012) found that inhibition of PKA could regulate the invasion and migration of human lung cancer cells. Therefore, PDE4B is likely to be a regulator of the occurrence and development of lung cancer.

### 4.4 Prostate gland

The prostate gland is located between the bladder and the original genital diaphragm and is a uniquely male organ (Prostate cancer, 2016). The altered genetic material triggered by chronic inflammation also promotes tumor transformation. Acute/chronic prostatitis in is one of the most prevalent diseases in adult men worldwide (De Marzo et al., 2007; Sfanos and De Marzo, 2012). The series of reactions triggered by prostate inflammation and the release of its other cytokines is one of the main causes of prostate cancer (PC) induction. Chronic prostatitis can make inflammatory cells infiltrate and subsequently worsen prostate disease and even develop into PC (Gurel et al., 2014). PC is the second most common malignant tumor in men worldwide, and patients with advanced PC almost always develop castration-resistant prostate cancer (CRPC), resulting in patient death (Siegel et al., 2022). It is worth noting that Rodrigo B de Alexandre et al. found that low expression of PDE4B in advanced PC, which is contrary to the high expression of PDE4B in other cancers, should be taken seriously (Azevedo et al., 2014). Eiji Kashiwagi et al. (2012) found that PDE4B downregulation leads to activation of the PKA signaling pathway. Studies have shown that the PKA signaling pathway was a key mediator of cell proliferation and differentiation in various normal and cancer cells, and oxidative stress in tumor cells inhibited PDE4B expression and activated PKA path. The PDE4B/PKA signaling pathway contributes to androgen-dependent prostate cancer progression to PC. This suggests that PDE4B is likely to be a potential target for the treatment of PC.

## 5 Regulation mechanisms of phosphodiesterase 4B in inflammation-mediated human disease

**FIGURE 2:**
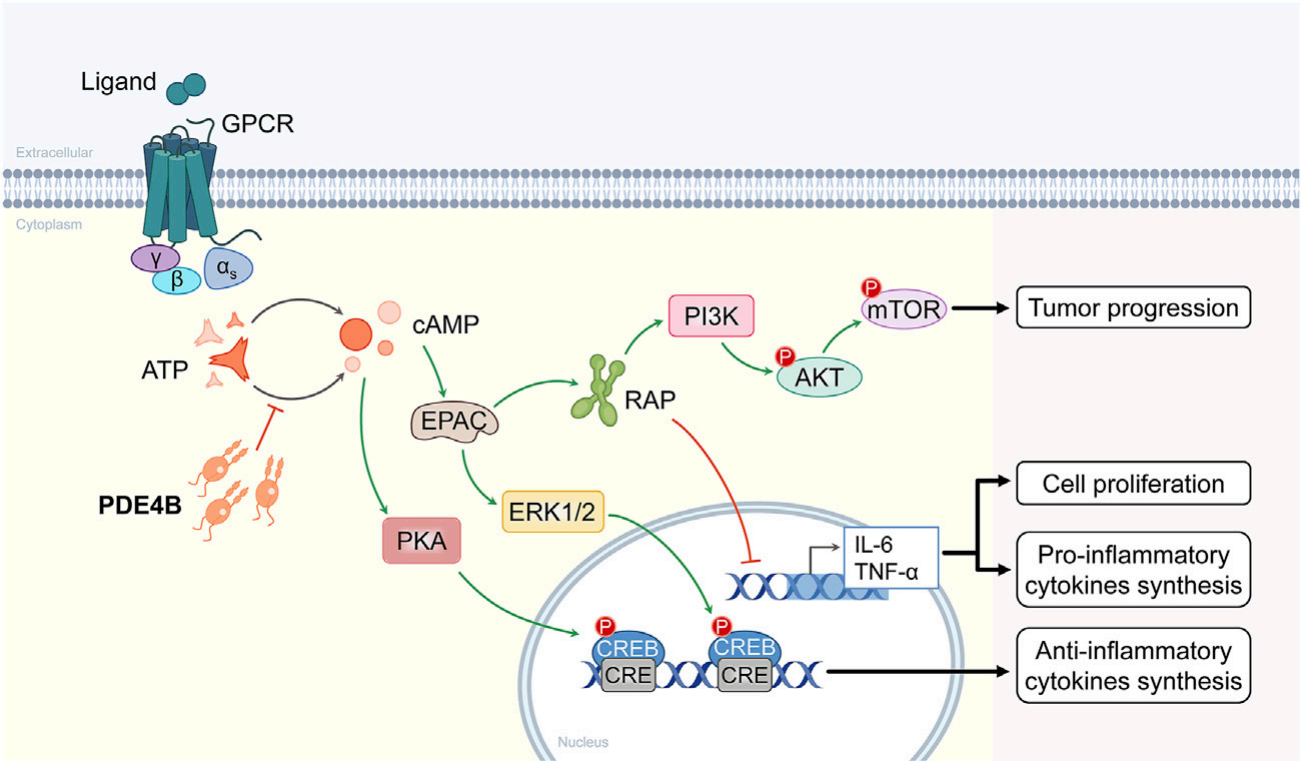
Molecular mechanisms of the signaling pathways involved in PDE4B. PDE4B is involved in the PI3K/AKT/mTOR signaling pathway and the other is through specific hydrolysis of cAMP followed by activation of the PKA signaling pathway.

PDE4s are the predominant cAMP degrading isozymes in most immune and inflammatory cells, the PDE4B isoform is expressed in a variety of immune and inflammatory cells, differentially regulated by various inflammatory stimuli (Peter et al., 2007). In addition, LPS has been shown to selectively induce PDE4 expression, which is consistent with elevated PDE4B expression in a variety of tumors (Jin and Conti, 2002). Numerous studies have shown that PDE4B played an important role in cancer progression. PDE4B participates in tumorigenesis and development mostly through two signaling pathways, one is through the PI3K/AKT/mTOR signaling pathway, and the other is through the specific hydrolysis of cAMP, which activates the PKA signaling pathway (Figure 2).

The PI3K/AKT/mTOR signaling pathway targets are among the most frequently activated signaling pathways in human cancers, and inhibitors represented by this signaling pathway have been successfully used for the treatment of primary and metastatic colorectal cancer (Narayanankutty, 2019), and anti-lung cancer drug development has been in the early trial stage (Tan, 2020). The PIK3CA gene is aberrantly expressed in human tumors. Akt is downstream of PI3K, and overexpression and mutation of its multiple isoforms are commonly associated with human disease processes. Overactivation of PI3K/AKT/mTOR signaling pathway has been found to be commonly associated with epidermal growth factor receptor (EGFR) mediated resistance to endocrine therapy and targeted therapies (Lian et al., 2019). Summarizing all previous studies, cAMP/PDE4B may regulate mTOR signaling pathway by modulating PI3K/AKT activity. Based on these observations, more attention should be paid to the progress of PDE4B inhibitors in the medical field.

In the absence of cAMP, PKA is an enzymatically inactive tetrameric holoenzyme consisting of two catalytic subunits dimerized with regulatory subunits. The PKA signaling pathway was a key mediator of cell proliferation and differentiation in various normal and cancer cells. Previous results have shown that the cAMP/PKA signaling pathway is activated by many different receptors and then coupled with G proteins to participate in signal transduction at the cell membrane (Hoddah et al., 2009). G proteins interact with the endosomal surface and are involved in the secretion of cAMP. In addition, PKA overexpression is associated with poor prognosis in several tumor types, including colorectal, breast and prostate cancers. Inhibition of PKA prevents proliferation and progression of lung and prostate tumor cells (Skoda et al., 2018). The above studies found that PDE4B specifically hydrolyzes cAMP, activates the PKA signaling pathway, and promotes prostate cancer development.

Overall, PDE4B is involved in the regulation of cellular signaling pathways and plays an important role in the development and clinical manifestations of inflammation as well as inflammation-induced cancer in multiple organs, suggesting PDE4B as a potential target for clinical therapy.

## 6 Future expectations

The results of previous studies have demonstrated that high expression of PDE4B was involved in inflammatory processes in several organs of the body and thus in the development of cancer, including hematologic malignancies (Kim et al., 2019), colon (Komatsu et al., 2013), and lung (Azam and Tripuraneni, 2014), and related results have demonstrated the important role of PDE4B in inflammation (Ma et al., 2014) (Table 1). With in-depth studies, the more attention was paid to the role of PDE4B in inflammatory. PDE4 inhibitors have been found to treat a variety of inflammatory diseases; for example, roflumilast can be used to block the inflammatory response and has a wide range of anti-inflammatory effects. Cyclamides (Zheng et al., 2019), dazinone derivatives (Allart-Simon et al., 2021), and triazolamide derivatives can inhibit the synthesis of PDE4 and subsequently exert anti-inflammatory effects, but PDE4B inhibitors, as highly selective PDE inhibitors, have a therapeutic function in inflammation while mitigating the side effects associated with the drugs. In addition, PDE4B was also found to have a positive effect on hematological tumor (Nagy et al., 2013), colon cancer (Nishi et al., 2017), lung cancer (Pullamsetti et al., 2013), liver cancer (Ding et al., 2012), etc. The only special case so far is the reduced expression of PDE4B in prostate cancer (Henderson et al., 2014). High expression levels of PDE4B promote the development of certain cancers and their subsequent invasion and metastasis. In addition, numerous studies have shown that PDE4B may be an oncogene in certain cancers and is closely associated with cancer pathogenesis through certain signaling pathways. Therefore, it is expected that PDE4B may be a potential target for cancer therapy. This section combines the latest technologies and research results in life sciences and medicine to look into the future of PDE4B. Cancer is one of the leading causes of death worldwide, and researchers are committed to investigate new therapeutic approaches. In recent years, many treatment concepts have emerged in addition to traditional radiotherapy and chemotherapy. Researchers have shifted the direction of treatment to the genetic level, the underlying mechanism of cancer pathogenesis, in order to improve the treatment outcome and prognosis of cancer. In the past, the traditional treatments for tumors were mostly surgery, radiotherapy, and chemotherapy. Previously, RNA was often used in basic experiments to interfere with the expression of target genes in order to achieve targeted regulation of genes. Since 2001, the huge project of sequencing the human genome, together with the development of CRISPR-Cas9 technology, gene editing is expected to become a viable biomedical tool (Cheng et al., 2020). In summary, PDE4B can not only be the focus of life science and medical research, but also can be connected with many other cutting-edge research results in related fields, and PDE4B can undoubtedly be a promising research direction.

**TABLE 1:** The relationship between abnormal PDE4B expression and inflammatory processes demonstrates the important role of PDE4B in inflammation.

Cancer type	Expression	Function	Refernces(PMID)
Diffuse large B-cell lymphoma (DLBCL)	+	Phosphodiesterase PDE4B restricts cAMP-associated PI3K/AKT-dependent apoptosis in diffuse large B-cell lymphoma	15331441
Acute Lymphoblastic Leukemia (ALL)	+	The effect of PDE4B on the treatment junction of ALL may be related to its overexpression and glucocorticoid resistance	35266293
Colorectal Cancer (CCa)	+	PDE4B can show tumor suppressive effects by inhibiting the mTOR-Myc axis	30528730
Lung Cancer (LUNG)	+	PDE4B is involved in the development of lung Cancer by affecting the cAMP-dependent protein kinase (PKA) activity	22954688
Prostate Cancer (PCa)	—	PDE4B downregulation leads to activation of the PKApathway pathway, and oxidative stress in tumor cells inhibits PDE4B expression and activates the PKA pathway pathway, thereby inhibiting prostate development	22529021

Accumulating evidence suggests that PDE4B plays an important role in the pathogenesis and clinical manifestations of cancer, including proliferation, migration, and drug resistance. In addition, PDE4B is involved in the regulation of multiple signaling pathways in cancer cells. For example, PDE4B can act as a target that modulates cAMP signaling pathway and plays a key role in maintaining the stemness of ovarian cancer (Huang et al., 2020), while cAMP/PDE4B signaling pathway can also modulate the malignant phenotype of CRC cells (Kim et al., 2019). PDE4B promotes melanoma invasion and metastasis by inhibiting the cAMP signaling pathway. PDE4B promotes cancer progression in diffuse large B-cell lymphoma (Smith et al., 2005; Suhasini et al., 2016), colorectal cancer (Pleiman et al., 2018), breast cancer (Luo et al., 2021), lymphoid carcinoma (Nagy et al., 2013) and liver cancer (Ding et al., 2012; Sung et al., 2012), whereas in prostate cancer (Kashiwagi et al., 2012), down-regulation of PDE4B contributes to the occurrence and development of prostate cancer. There is growing evidence that increasing intracellular cAMP levels may be one way to improve chronic inflammation. One of the means of increasing the level of cAMP is to inhibit its degradation, from which small molecule inhibitors of PDE4 were developed. PDE4 inhibitors have been found to reduce the level of inflammatory response for the treatment of inflammatory bowel disease, atopic dermatitis, rheumatoid arthritis and other diseases, such as Apt can be used for psoriatic arthritis, and rofluskast can be used to treat asthma. A series of PDE4 inhibitors, such as roromeste, oglemilast, GSK256066, CHF6001, YM976, GS-5759, etc., have been in development to improve the selectivity of drugs to reduce adverse reactions, such as inhibitors that specifically target PDE4B in the treatment of inflammation (Tralau-Stewart et al., 2011), colorectal diseases, and cancer (Nose et al., 2016)have shown a promising therapeutic future.

## 7 Conclusion

In summary, PDE4B is involved in several mechanisms of the organism, and from laboratory discovery to clinical application, PDE4B has shown its potential application. In future studies, with the increase of clinical sample size and the clarification of PDE4B-related regulatory mechanisms, PDE4B will definitely provide new ideas for the diagnosis and treatment of human diseases.

## Abbreviations

ALL, Acute Lymphoblastic Leukemia; cAMP, cyclic AMP; CRC, Colorectal cancer; cGMP, cyclic guanosine monophosphate; CRPC, castration-resistant prostate cancer; DLBCL, Diffuse large B-cell lymphoma; EGFR, epidermal growth factor receptor; IACR, international agency for research on cancer; LPS, lipopolysaccharide; NSCLC, Non-small cell lung cancer; NF-κB, nuclear factor kappa-B; PDE4B, phosphodiesterase 4B; PDEs, phosphodiesteras; PDE4A, phosphodiesterase 4A; PDE4D, phosphodiesterase 4D; PKA, protein kinase A; ROS, reactive oxygen species; PC, prostate cancer.

## References

[B1] Ahlström M. Pekkinen M. Huttunen M. Lamberg-Allardt C. (2005). Dexamethasone down-regulates cAMP-phosphodiesterase in human osteosarcoma cells. Biochem. Pharmacol. 69 (2), 267–275. 10.1016/j.bcp.2004.09.012 15627479

[B2] Ahmad F. Murata T. Shimizu K. Degerman E. Maurice D. Manganiello V. (2015). Cyclic nucleotide phosphodiesterases: Important signaling modulators and therapeutic targets. Oral Dis. 21 (1), e25–e50. 10.1111/odi.12275 25056711 PMC4275405

[B3] Allart-Simon I. Moniot A. Bisi N. Ponce-Vargas M. Audonnet S. Laronze-Cochard M. (2021). Pyridazinone derivatives as potential anti-inflammatory agents: Synthesis and biological evaluation as PDE4 inhibitors. RSC Med. Chem. 12 (4), 584–592. 10.1039/d0md00423e 34046629 PMC8127987

[B4] Annibali O. Hohaus S. Marchesi F. Cantonetti M. Di Rocco A. Tomarchio V. (2019). The neutrophil/lymphocyte ratio ≥3.5 is a prognostic marker in diffuse large B-cell lymphoma: A retrospective analysis from the database of the Italian regional network 'rete ematologica del lazio per i linfomi' (RELLI). Leuk. Lymphoma 60 (14), 3386–3394. 10.1080/10428194.2019.1633628 31259651

[B5] Ariffin H. Azanan M. S. Abd Ghafar S. S. Oh L. Lau K. H. Thirunavakarasu T. (2017). Young adult survivors of childhood acute lymphoblastic leukemia show evidence of chronic inflammation and cellular aging. Cancer 123 (21), 4207–4214. 10.1002/cncr.30857 28654149

[B6] Azam M. A. Tripuraneni N. S. (2014). Selective phosphodiesterase 4B inhibitors: A review. Sci. Pharm. 82 (3), 453–481. 10.3797/scipharm.1404-08 25853062 PMC4318138

[B7] Azevedo M. F. Faucz F. R. Bimpaki E. Horvath A. Levy I. de Alexandre R. B. (2014). Clinical and molecular genetics of the phosphodiesterases (PDEs). Endocr. Rev. 35 (2), 195–233. 10.1210/er.2013-1053 24311737 PMC3963262

[B8] Bender A. T. Beavo J. A. (2006). Cyclic nucleotide phosphodiesterases: Molecular regulation to clinical use. Pharmacol. Rev. 58 (3), 488–520. 10.1124/pr.58.3.5 16968949

[B9] Biller L. H. Schrag D. (2021). Diagnosis and treatment of metastatic colorectal cancer: A review. Jama 325 (7), 669–685. 10.1001/jama.2021.0106 33591350

[B10] Blommaert D. Sergeant N. Delehedde M. Jouy N. Mitchell V. Franck T. (2019). Expression, localization, and concentration of A-kinase anchor protein 4 (AKAP4) and its precursor (proAKAP4) in equine semen: Promising marker correlated to the total and progressive motility in thawed spermatozoa. Theriogenology 131, 52–60. 10.1016/j.theriogenology.2019.03.011 30947075

[B11] Bolger G. B. (2017). The PDE4 cAMP-specific phosphodiesterases: Targets for drugs with antidepressant and memory-enhancing action. Adv. Neurobiol. 17, 63–102. 10.1007/978-3-319-58811-7_4 28956330

[B12] Cai J. Harrison A. P. Zheng Y. Yan K. Huo Y. Xiao J. (2021). Lesion-Harvester: Iteratively mining unlabeled lesions and hard-negative examples at scale. IEEE Trans. Med. Imaging 40 (1), 59–70. 10.1109/TMI.2020.3022034 32894709

[B13] Campbell S. L. van Groen T. Kadish I. Smoot L. H. M. Bolger G. B. (2017). Altered phosphorylation, electrophysiology, and behavior on attenuation of PDE4B action in hippocampus. BMC Neurosci. 18 (1), 77. 10.1186/s12868-017-0396-6 29197324 PMC5712142

[B14] Chen E. J. Chen S. Zhou F. L. (2021). Mechanism of TRIM27 promoting inflammatory response in lung cancer cells. Zhonghua Zhong Liu Za Zhi 43 (10), 1076–1081. 10.3760/cma.j.cn112152-20191204-00784 34695898

[B15] Chen Y. Jiang P. Wen J. Wu Z. Li J. Chen Y. (2020). Integrated bioinformatics analysis of the crucial candidate genes and pathways associated with glucocorticoid resistance in acute lymphoblastic leukemia. Cancer Med. 9 (8), 2918–2929. 10.1002/cam4.2934 32096603 PMC7163086

[B16] Cheng X. Fan S. Wen C. Du X. (2020). CRISPR/Cas9 for cancer treatment: Technology, clinical applications and challenges. Brief. Funct. Genomics 19 (3), 209–214. 10.1093/bfgp/elaa001 32052006

[B17] Coussens L. M. Werb Z. (2002). Inflammation and cancer. Nature 420 (6917), 860–867. 10.1038/nature01322 12490959 PMC2803035

[B18] Dagenais G. R. Leong D. P. Rangarajan S. Lanas F. Lopez-Jaramillo P. Gupta R. (2020). Variations in common diseases, hospital admissions, and deaths in middle-aged adults in 21 countries from five continents (PURE): A prospective cohort study. Lancet 395 (10226), 785–794. 10.1016/S0140-6736(19)32007-0 31492501

[B19] De Marzo A. M. Platz E. A. Sutcliffe S. Xu J. Grönberg H. Drake C. G. (2007). Inflammation in prostate carcinogenesis. Nat. Rev. Cancer 7 (4), 256–269. 10.1038/nrc2090 17384581 PMC3552388

[B20] Diakos C. I. Charles K. A. McMillan D. C. Clarke S. J. (2014). Cancer-related inflammation and treatment effectiveness. Lancet. Oncol. 15 (11), e493–e503. 10.1016/S1470-2045(14)70263-3 25281468

[B21] Ding D. Lou X. Hua D. Yu W. Li L. Wang J. (2012). Recurrent targeted genes of Hepatitis B virus in the liver cancer genomes identified by a next-generation sequencing-based approach. PLoS Genet. 8 (12), e1003065. 10.1371/journal.pgen.1003065 23236287 PMC3516541

[B22] Diori Karidio I. Sanlier S. H. (2021). Reviewing cancer's biology: An eclectic approach. J. Egypt. Natl. Canc. Inst. 33 (1), 32. 10.1186/s43046-021-00088-y 34719756 PMC13316913

[B23] Fortin M. D'Anjou H. Higgins M. E. Gougeon J. Aubé P. Moktefi K. (2009). A multi-target antisense approach against PDE4 and PDE7 reduces smoke-induced lung inflammation in mice. Respir. Res. 10 (1), 39. 10.1186/1465-9921-10-39 19457265 PMC2696437

[B24] Gleeson M. Bishop N. C. Stensel D. J. Lindley M. R. Mastana S. S. Nimmo M. A. (2011). The anti-inflammatory effects of exercise: Mechanisms and implications for the prevention and treatment of disease. Nat. Rev. Immunol. 11 (9), 607–615. 10.1038/nri3041 21818123

[B25] Greten F. R. Grivennikov S. I. (2019). Inflammation and cancer: Triggers, mechanisms, and consequences. Immunity 51 (1), 27–41. 10.1016/j.immuni.2019.06.025 31315034 PMC6831096

[B26] Guo B. Lager K. M. Henningson J. N. Miller L. C. Schlink S. N. Kappes M. A. (2013). Experimental infection of United States swine with a Chinese highly pathogenic strain of porcine reproductive and respiratory syndrome virus. Virology 435 (2), 372–384. 10.1016/j.virol.2012.09.013 23079105 PMC7111980

[B27] Gurel B. Lucia M. S. Thompson I. M. Jr. Goodman P. J. Tangen C. M. Kristal A. R. (2014). Chronic inflammation in benign prostate tissue is associated with high-grade prostate cancer in the placebo arm of the prostate cancer prevention trial. Cancer Epidemiol. Biomarkers Prev. 23 (5), 847–856. 10.1158/1055-9965.EPI-13-1126 24748218 PMC4012292

[B28] He N. Kim N. Song M. Park C. Kim S. Park E. Y. (2014). Integrated analysis of transcriptomes of cancer cell lines and patient samples reveals STK11/LKB1-driven regulation of cAMP phosphodiesterase-4D. Mol. Cancer Ther. 13 (10), 2463–2473. 10.1158/1535-7163.MCT-14-0297 25122068

[B29] Henderson D. J. Byrne A. Dulla K. Jenster G. Hoffmann R. Baillie G. S. (2014). The cAMP phosphodiesterase-4D7 (PDE4D7) is downregulated in androgen-independent prostate cancer cells and mediates proliferation by compartmentalising cAMP at the plasma membrane of VCaP prostate cancer cells. Br. J. Cancer 110 (5), 1278–1287. 10.1038/bjc.2014.22 24518597 PMC3950871

[B30] Hoddah H. Marcantoni A. Comunanza V. Carabelli V. Carbone E. (2009). L-type channel inhibition by CB1 cannabinoid receptors is mediated by PTX-sensitive G proteins and cAMP/PKA in GT1-7 hypothalamic neurons. Cell Calcium 46 (5-6), 303–312. 10.1016/j.ceca.2009.08.007 19818494

[B31] Hsien Lai S. Zervoudakis G. Chou J. Gurney M. E. Quesnelle K. M. (2020). PDE4 subtypes in cancer. Oncogene 39 (19), 3791–3802. 10.1038/s41388-020-1258-8 32203163 PMC7444459

[B32] Huang H. Wang Y. Kandpal M. Zhao G. Cardenas H. Ji Y. (2020). FTO-dependent N (6)-methyladenosine modifications inhibit ovarian cancer stem cell self-renewal by blocking cAMP signaling. Cancer Res. 80 (16), 3200–3214. 10.1158/0008-5472.CAN-19-4044 32606006 PMC7442742

[B33] Jacob C. Martin-Chouly C. Lagente V. (2002). Type 4 phosphodiesterase-dependent pathways: Role in inflammatory processes. Therapie 57 (2), 163–168. 12185965

[B34] Jacobs K. Kligerman S. (2019). Immune-mediated lung diseases. Semin. Ultrasound CT MR 40 (3), 213–228. 10.1053/j.sult.2018.11.011 31200870

[B35] Jiang X. Paskind M. Weltzien R. Epstein P. M. (1998). Expression and regulation of mRNA for distinct isoforms of cAMP-specific PDE-4 in mitogen-stimulated and leukemic human lymphocytes. Cell biochem. Biophys. 28 (2-3), 135–160. 10.1007/BF02737809 9515164

[B36] Jin S. L. Conti M. (2002). Induction of the cyclic nucleotide phosphodiesterase PDE4B is essential for LPS-activated TNF-alpha responses. Proc. Natl. Acad. Sci. U. S. A. 99 (11), 7628–7633. 10.1073/pnas.122041599 12032334 PMC124305

[B37] Jin S. L. Lan L. Zoudilova M. Conti M. (2005). Specific role of phosphodiesterase 4B in lipopolysaccharide-induced signaling in mouse macrophages. J. Immunol. 175 (3), 1523–1531. 10.4049/jimmunol.175.3.1523 16034090

[B38] Kachuri L. Johansson M. Rashkin S. R. Graff R. E. Bossé Y. Manem V. (2020). Immune-mediated genetic pathways resulting in pulmonary function impairment increase lung cancer susceptibility. Nat. Commun. 11 (1), 27. 10.1038/s41467-019-13855-2 31911640 PMC6946810

[B39] Kashiwagi E. Shiota M. Yokomizo A. Itsumi M. Inokuchi J. Uchiumi T. (2012). Downregulation of phosphodiesterase 4B (PDE4B) activates protein kinase A and contributes to the progression of prostate cancer. Prostate 72 (7), 741–751. 10.1002/pros.21478 22529021

[B40] Kay J. Thadhani E. Samson L. Engelward B. (2019). Inflammation-induced DNA damage, mutations and cancer. DNA Repair (Amst) 83, 102673. 10.1016/j.dnarep.2019.102673 31387777 PMC6801086

[B41] Kim D. U. Kwak B. Kim S. W. (2019). Phosphodiesterase 4B is an effective therapeutic target in colorectal cancer. Biochem. Biophys. Res. Commun. 508 (3), 825–831. 10.1016/j.bbrc.2018.12.004 30528730

[B42] Kim J. Jeong D. Nam J. Aung T. N. Gim J. A. Park K. U. (2015). MicroRNA-124 regulates glucocorticoid sensitivity by targeting phosphodiesterase 4B in diffuse large B cell lymphoma. Gene 558 (1), 173–180. 10.1016/j.gene.2015.01.001 25576220

[B43] Kim S. W. Rai D. McKeller M. R. Aguiar R. C. (2009). Rational combined targeting of phosphodiesterase 4B and SYK in DLBCL. Blood 113 (24), 6153–6160. 10.1182/blood-2009-02-206128 19369227 PMC2699235

[B44] Kolosionek E. Savai R. Ghofrani H. A. Weissmann N. Guenther A. Grimminger F. (2009). Expression and activity of phosphodiesterase isoforms during epithelial mesenchymal transition: The role of phosphodiesterase 4. Mol. Biol. Cell 20 (22), 4751–4765. 10.1091/mbc.e09-01-0019 19759179 PMC2777105

[B45] Komatsu K. Lee J. Y. Miyata M. Hyang Lim J. Jono H. Koga T. (2013). Inhibition of PDE4B suppresses inflammation by increasing expression of the deubiquitinase CYLD. Nat. Commun. 4, 1684. 10.1038/ncomms2674 23575688 PMC3644066

[B46] Korniluk A. Koper O. Kemona H. Dymicka-Piekarska V. (2017). From inflammation to cancer. Ir. J. Med. Sci. 186 (1), 57–62. 10.1007/s11845-016-1464-0 27156054 PMC5323483

[B47] Kumar S. Sharawat S. K. (2018). Epigenetic regulators of programmed death-ligand 1 expression in human cancers. Transl. Res. 202, 129–145. 10.1016/j.trsl.2018.05.011 30401465

[B48] Li H. Lan T. Liu H. Liu C. Dai J. Xu L. (2022). IL-6-induced cGGNBP2 encodes a protein to promote cell growth and metastasis in intrahepatic cholangiocarcinoma. Hepatology 75 (6), 1402–1419. 10.1002/hep.32232 34758510 PMC9306806

[B49] Lian G. Chen S. Ouyang M. Li F. Chen L. Yang J. (2019). Colon cancer cell secretes EGF to promote M2 polarization of TAM through EGFR/PI3K/AKT/mTOR pathway. Technol. Cancer Res. Treat. 18, 1533033819849068. 10.1177/1533033819849068 31088266 PMC6535704

[B50] Liu C. Shi K. (2021). A review on methodology in O(3)-NOx-VOC sensitivity study. Environ. Pollut. 291, 118249. 10.1016/j.envpol.2021.118249 34600066

[B51] Luo R. Chong W. Wei Q. Zhang Z. Wang C. Ye Z. (2021). Whole-exome sequencing identifies somatic mutations and intratumor heterogeneity in inflammatory breast cancer. NPJ Breast Cancer 7 (1), 72. 10.1038/s41523-021-00278-w 34075047 PMC8169683

[B52] Ma H. Shi J. Wang C. Guo L. Gong Y. Li J. (2014). Blockade of PDE4B limits lung vascular permeability and lung inflammation in LPS-induced acute lung injury. Biochem. Biophys. Res. Commun. 450 (4), 1560–1567. 10.1016/j.bbrc.2014.07.024 25019986

[B53] Mahmood B. Damm M. M. Jensen T. S. Backe M. B. Dahllöf M. S. Poulsen S. S. (2016). Phosphodiesterases in non-neoplastic appearing colonic mucosa from patients with colorectal neoplasia. BMC cancer 16 (1), 938. 10.1186/s12885-016-2980-z 27927168 PMC5141637

[B54] McGranahan N. Swanton C. (2017). Cancer evolution constrained by the immune microenvironment. Cell 170 (5), 825–827. 10.1016/j.cell.2017.08.012 28841415

[B55] Medinger M. Heim D. Lengerke C. Halter J. P. Passweg J. R. (2019). Acute lymphoblastic leukemia - diagnosis and therapy. Ther. Umsch. 76 (9), 510–515. 10.1024/0040-5930/a001127 32157966

[B56] Meira L. B. Bugni J. M. Green S. L. Lee C. W. Pang B. Borenshtein D. (2008). DNA damage induced by chronic inflammation contributes to colon carcinogenesis in mice. J. Clin. Invest. 118 (7), 2516–2525. 10.1172/JCI35073 18521188 PMC2423313

[B57] Moon E. Lee R. Near R. Weintraub L. Wolda S. Lerner A. (2002). Inhibition of PDE3B augments PDE4 inhibitor-induced apoptosis in a subset of patients with chronic lymphocytic leukemia. Clin. Cancer Res. 8 (2), 589–595. 11839681

[B58] Moreira R. G. Saraiva-Duarte J. M. Pereira A. C. Sosa-Macias M. Galaviz-Hernandez C. Santolalla M. L. (2022). Population genetics of PDE4B (phosphodiesterase-4B) in neglected Native Americans: Implications for cancer pharmacogenetics. Clin. Transl. Sci. 15, 1400–1405. 10.1111/cts.13266 35266293 PMC9199872

[B59] Nagy Z. S. Ross J. A. Rodriguez G. Balint B. L. Szeles L. Nagy L. (2013). Genome wide mapping reveals PDE4B as an IL-2 induced STAT5 target gene in activated human PBMCs and lymphoid cancer cells. PLoS One 8 (2), e57326. 10.1371/journal.pone.0057326 23451206 PMC3581501

[B60] Nam J. Kim D. U. Kim E. Kwak B. Ko M. J. Oh A. Y. (2019). Disruption of the Myc-PDE4B regulatory circuitry impairs B-cell lymphoma survival. Leukemia 33 (12), 2912–2923. 10.1038/s41375-019-0492-y 31138843

[B61] Narayanankutty A. (2019). PI3K/akt/mTOR pathway as a therapeutic target for colorectal cancer: A review of preclinical and clinical evidence. Curr. Drug Targets 20 (12), 1217–1226. 10.2174/1389450120666190618123846 31215384

[B62] Nishi K. Luo H. Ishikura S. Doi K. Iwaihara Y. Wills L. (2017). Apremilast induces apoptosis of human colorectal cancer cells with mutant KRAS. Anticancer Res. 37 (7), 3833–3839. 10.21873/anticanres.11762 28668883

[B63] Nose T. Kondo M. Shimizu M. Hamura H. Yamaguchi Y. Sekine T. (2016). Pharmacological profile of GPD-1116, an inhibitor of phosphodiesterase 4. Biol. Pharm. Bull. 39 (5), 689–698. 10.1248/bpb.b15-00652 27150141

[B64] Ocana A. Nieto-Jiménez C. Pandiella A. Templeton A. J. (2017). Neutrophils in cancer: Prognostic role and therapeutic strategies. Mol. Cancer 16 (1), 137. 10.1186/s12943-017-0707-7 28810877 PMC5558711

[B65] Peter D. Jin S. L. Conti M. Hatzelmann A. Zitt C. (2007). Differential expression and function of phosphodiesterase 4 (PDE4) subtypes in human primary CD4+ T cells: Predominant role of PDE4D. J. Immunol. 178 (8), 4820–4831. 10.4049/jimmunol.178.8.4820 17404263

[B66] Pleiman J. K. Irving A. A. Wang Z. Toraason E. Clipson L. Dove W. F. (2018). The conserved protective cyclic AMP-phosphodiesterase function PDE4B is expressed in the adenoma and adjacent normal colonic epithelium of mammals and silenced in colorectal cancer. PLoS Genet. 14 (9), e1007611. 10.1371/journal.pgen.1007611 30188895 PMC6143270

[B67] Prostate cancer (2016). Nursing standard. london: Royal College of Nursing Great Britain, 17–30.

[B68] Pullamsetti S. S. Banat G. A. Schmall A. Szibor M. Pomagruk D. Hänze J. (2013). Phosphodiesterase-4 promotes proliferation and angiogenesis of lung cancer by crosstalk with HIF. Oncogene 32 (9), 1121–1134. 10.1038/onc.2012.136 22525277

[B69] Quail D. F. Joyce J. A. (2013). Microenvironmental regulation of tumor progression and metastasis. Nat. Med. 19 (11), 1423–1437. 10.1038/nm.3394 24202395 PMC3954707

[B70] Rahamim Ben-Navi L. Almog T. Yao Z. Seger R. Naor Z. (2016). A-Kinase Anchoring Protein 4 (AKAP4) is an ERK1/2 substrate and a switch molecule between cAMP/PKA and PKC/ERK1/2 in human spermatozoa. Sci. Rep. 6, 37922. 10.1038/srep37922 27901058 PMC5128789

[B71] Rambaldi A. Boschini C. Gritti G. Delaini F. Oldani E. Rossi A. (2013). The lymphocyte to monocyte ratio improves the IPI-risk definition of diffuse large B-cell lymphoma when rituximab is added to chemotherapy. Am. J. Hematol. 88 (12), 1062–1067. 10.1002/ajh.23566 23940056

[B72] Rickles R. J. Pierce L. T. Giordano T. P. 3rd Tam W. F. McMillin D. W. Delmore J. (2010). Adenosine A2A receptor agonists and PDE inhibitors: A synergistic multitarget mechanism discovered through systematic combination screening in B-cell malignancies. Blood 116 (4), 593–602. 10.1182/blood-2009-11-252668 20382846

[B73] Schäfer M. Werner S. (2008). Cancer as an overhealing wound: An old hypothesis revisited. Nat. Rev. Mol. Cell Biol. 9 (8), 628–638. 10.1038/nrm2455 18628784

[B74] Schick M. A. Schlegel N. (2022). Clinical implication of phosphodiesterase-4-inhibition. Int. J. Mol. Sci. 23 (3), 1209. 10.3390/ijms23031209 35163131 PMC8835523

[B75] Schmitz R. Wright G. W. Huang D. W. Johnson C. A. Phelan J. D. Wang J. Q. (2018). Genetics and pathogenesis of diffuse large B-cell lymphoma. N. Engl. J. Med. 378 (15), 1396–1407. 10.1056/NEJMoa1801445 29641966 PMC6010183

[B76] Sengupta R. Honey K. (2019). AACR cancer progress report 2019: Transforming lives through innovative cancer science. Clin. Cancer Res. 25 (18), 5431. 10.1158/1078-0432.CCR-19-2655 31581117

[B77] Sengupta R. Zaidi S. K. (2021). AACR cancer progress report 2021: Discovery science driving clinical breakthroughs. Clin. Cancer Res. 27 (21), 5757–5759. 10.1158/1078-0432.CCR-21-3367 34645645

[B78] Sfanos K. S. De Marzo A. M. (2012). Prostate cancer and inflammation: The evidence. Histopathology 60 (1), 199–215. 10.1111/j.1365-2559.2011.04033.x 22212087 PMC4029103

[B79] Sfanos K. S. Yegnasubramanian S. Nelson W. G. De Marzo A. M. (2018). The inflammatory microenvironment and microbiome in prostate cancer development. Nat. Rev. Urol. 15 (1), 11–24. 10.1038/nrurol.2017.167 29089606

[B80] Shaikh D. Zhou Q. Chen T. Ibe J. C. Raj J. U. Zhou G. (2012). cAMP-dependent protein kinase is essential for hypoxia-mediated epithelial-mesenchymal transition, migration, and invasion in lung cancer cells. Cell. Signal. 24 (12), 2396–2406. 10.1016/j.cellsig.2012.08.007 22954688

[B81] Siegel R. L. Miller K. D. Fuchs H. E. Jemal A. (2021). Cancer statistics, 2021. Ca. Cancer J. Clin. 71 (1), 7–33. 10.3322/caac.21654 33433946

[B82] Siegel R. L. Miller K. D. Fuchs H. E. Jemal A. (2022). Cancer statistics, 2022. Ca. Cancer J. Clin. 72 (1), 7–33. 10.3322/caac.21708 35020204

[B83] Singh N. Baby D. Rajguru J. P. Patil P. B. Thakkannavar S. S. Pujari V. B. (2019). Inflammation and cancer. Ann. Afr. Med. 18 (3), 121–126. 10.4103/aam.aam_56_18 31417011 PMC6704802

[B84] Skoda A. M. Simovic D. Karin V. Kardum V. Vranic S. Serman L. (2018). The role of the hedgehog signaling pathway in cancer: A comprehensive review. Bosn. J. Basic Med. Sci. 18 (1), 8–20. 10.17305/bjbms.2018.2756 29274272 PMC5826678

[B85] Smith P. G. Wang F. Wilkinson K. N. Savage K. J. Klein U. Neuberg D. S. (2005). The phosphodiesterase PDE4B limits cAMP-associated PI3K/AKT-dependent apoptosis in diffuse large B-cell lymphoma. Blood 105 (1), 308–316. 10.1182/blood-2004-01-0240 15331441

[B86] Smith S. M. Wachter K. Burris H. A. 3rd Schilsky R. L. George D. J. Peterson D. E. (2021). Clinical cancer advances 2021: ASCO's report on progress against cancer. J. Clin. Oncol. 39 (10), 1165–1184. 10.1200/JCO.20.03420 33527845

[B87] Suarez-Carmona M. Lesage J. Cataldo D. Gilles C. (2017). EMT and inflammation: Inseparable actors of cancer progression. Mol. Oncol. 11 (7), 805–823. 10.1002/1878-0261.12095 28599100 PMC5496491

[B88] Suhasini A. N. Wang L. Holder K. N. Lin A. P. Bhatnagar H. Kim S. W. (2016). A phosphodiesterase 4B-dependent interplay between tumor cells and the microenvironment regulates angiogenesis in B-cell lymphoma. Leukemia 30 (3), 617–626. 10.1038/leu.2015.302 26503641 PMC4775385

[B89] Sung W. K. Zheng H. Li S. Chen R. Liu X. Li Y. (2012). Genome-wide survey of recurrent HBV integration in hepatocellular carcinoma. Nat. Genet. 44 (7), 765–769. 10.1038/ng.2295 22634754

[B90] Tan A. C. (2020). Targeting the PI3K/Akt/mTOR pathway in non-small cell lung cancer (NSCLC). Thorac. Cancer 11 (3), 511–518. 10.1111/1759-7714.13328 31989769 PMC7049515

[B91] Tavares L. P. Negreiros-Lima G. L. Lima K. M. Pmr E. S. Pinho V. Teixeira M. M. (2020). Blame the signaling: Role of cAMP for the resolution of inflammation. Pharmacol. Res. 159, 105030. 10.1016/j.phrs.2020.105030 32562817

[B92] Thai A. A. Solomon B. J. Sequist L. V. Gainor J. F. Heist R. S. (2021). Lung cancer. Lancet 398 (10299), 535–554. 10.1016/S0140-6736(21)00312-3 34273294

[B93] Tralau-Stewart C. J. Williamson R. A. Nials A. T. Gascoigne M. Dawson J. Hart G. J. (2011). GSK256066, an exceptionally high-affinity and selective inhibitor of phosphodiesterase 4 suitable for administration by inhalation: *In vitro*, kinetic, and *in vivo* characterization. J. Pharmacol. Exp. Ther. 337 (1), 145–154. 10.1124/jpet.110.173690 21205923

[B94] Vineis P. Wild C. P. (2014). Global cancer patterns: Causes and prevention. Lancet 383 (9916), 549–557. 10.1016/S0140-6736(13)62224-2 24351322

[B95] Yan K. Wang X. Lu L. Summers R. M. (2018). DeepLesion: Automated mining of large-scale lesion annotations and universal lesion detection with deep learning. J. Med. Imaging 5 (3), 036501. 10.1117/1.JMI.5.3.036501 PMC6052252 30035154

[B96] Yashiro M. (2014). Ulcerative colitis-associated colorectal cancer. World J. Gastroenterol. 20 (44), 16389–16397. 10.3748/wjg.v20.i44.16389 25469007 PMC4248182

[B97] Zhang H. Kong Q. Wang J. Jiang Y. Hua H. (2020). Complex roles of cAMP-PKA-CREB signaling in cancer. Exp. Hematol. Oncol. 9 (1), 32. 10.1186/s40164-020-00191-1 33292604 PMC7684908

[B98] Zhao H. Wu L. Yan G. Chen Y. Zhou M. Wu Y. (2021). Inflammation and tumor progression: Signaling pathways and targeted intervention. Signal Transduct. Target. Ther. 6 (1), 263. 10.1038/s41392-021-00658-5 34248142 PMC8273155

[B99] Zhao S. Dong X. Shen W. Ye Z. Xiang R. (2016). Machine learning-based classification of diffuse large B-cell lymphoma patients by eight gene expression profiles. Cancer Med. 5 (5), 837–852. 10.1002/cam4.650 26869285 PMC4864813

[B100] Zheng X. Y. Chen J. C. Xie Q. M. Chen J. Q. Tang H. F. (2019). Anti-inflammatory effect of ciclamilast in an allergic model involving the expression of PDE4B. Mol. Med. Rep. 19 (3), 1728–1738. 10.3892/mmr.2019.9802 30628641

